# Mystery shopping in community drug shops: research as development in rural Tanzania

**DOI:** 10.1186/1475-2875-11-S1-O19

**Published:** 2012-10-15

**Authors:** Angel Dillip, Sandra Alba, Christopher Mshana, Manuell Hetze, Jafari Liana, Christian Lengeler, Iddy Mayumana, Alexander Schulze, Hassan Mshinda, Brigit Obrist

**Affiliations:** 1 Ifakara Health Institute, Off Mlabani Passage P.O.Box 53, Ifakara, Morogoro, Tanzania; 2 Swiss Tropical and Public Health Institute, Socinstrasse 57, CH-4002, Basel, Switzerland; 3 University of Basel, Petersplatz 1, 4003 Basel, Switzerland; 4 Papua New Guinea Institute of Medical Research, Goroka, EHP 441, Papua New Guinea; 5 Management Sciences for Health, Dares Salaam, Tanzania; 6 Novartis Foundation for Sustainable Development, WRO-1002.11.56, 4002 Basel, Switzerland; 7 Tanzania Commission for Science and Technology, P.O. Box 4302, Dar es Salaam, Tanzania; 8 Institute of Social Anthropology, University of Basel, Münsterplatz 19, Basel, Switzerland

## Background

Throughout Africa, the private sector plays an important role in malaria treatment complementing formal health services. However this sector is faced by a number of challenges including poor dispensing practices by unqualified staff. The Accredited Drug Dispensing Outlet (ADDO) program was introduced in Tanzania in 2002 to improve the quality of retail services and especially of dispensing practices. The study adapted the often contested mystery shopping methodology and trained local community members to assess practices of ADDO dispensers. The study then compared the assessed dispensers’ practices before and after ADDO interventions.

## Methods

Mystery shoppers were identified in the villages with the assistance of Health Demographic Surveillance System field staff. A total of 865 visits were made to general shops and drug shops between 2004 and 2009. Three case scenarios were developed to assess the quality of treatment; a) child aged 2 - 4 months, with fever/hot body for one day and problems with drinking/breastfeeding, b) child aged 2 - 4 years, with recurring fever/hot body for 3 days problems with drinking, eating, diarrhoea and tiredness/not playing as usual and c) adult, with recurring fever/hot body for 2 days, headache, dizziness and loss of appetite.

## Results

Study findings indicate improvements in dispensers’knowledge and practices in management of fever, especially after the roll out of ADDO program in the study area. A 30 percent increase was noted after ADDO interventions on four assessed indicators developed based on the national malaria control guideline on malaria case management. On the other hand advice on the use of Insecticide Treated Nets as a measure to prevent malaria was not consistent over years even after ADDO interventions. Children aged two to four years and adults were more likely to be provided with anti-malarials than children between two to four months. Despite challenges posed against the methodology, findings reveals how useful the mystery shopping technique can be for community assessments of ADDO interventions in retail outlets.

## Conclusion

Study findings signify the importance of ADDO interventions in improving malaria case management in drug retail outlets. If ADDOs are closely monitored and strengthened to provide appropriate malaria treatment and the program is rolled throughout the country, a reduction in malaria morbidity and mortality is possible in the country. Innovative community based participatory research approaches and more systematic mystery shopping techniques would allow for comparative community-based assessments of ADDO interventions across regions.

